# Colon-Specific Drug Delivery Behavior of pH-Responsive PMAA/Perlite Composite

**DOI:** 10.3390/ijms11041546

**Published:** 2010-04-12

**Authors:** Mehrdad Mahkam, Laleh Vakhshouri

**Affiliations:** Chemistry Department, Azarbaijan University of Tarbiat Moallem, Tabriz, Iran

**Keywords:** methacrylic acid, perlite, silylation, polymerization, polymer grafting

## Abstract

The preparation, characterization, and *in vitro* release of 5-aminosalicylic acid (5-ASA) from methacrylic acid (MAA)/perlite composites (APC) prepared via a sol–gel route are reported. The free-radical graft polymerization of methacrylic acid (MAA) onto perlite particles was studied experimentally. The grafting procedure consisted of surface activation with 3-(trimethoxysilyl) propyl methacrylate (TSPA), followed by free-radical graft polymerization of methacrylic acid (MAA) in ethyl acetate with 2,2′-azobisisobutyronitrile (AIBN) initiator. The composition of the composites hybrid materials was determined by FTIR spectroscopy. Equilibrium swelling studies were carried out in enzyme-free simulated gastric and intestinal fluids (SGF and SIF, respectively). The dried composites were immersed in a saturated solution of 5-ASA in water overnight and dried over a period of three days at room temperature and the *in vitro* release profiles were established separately in both (SGF, pH 1) and (SIF, pH 7.4). The 5-ASA concentration of the solution was measured using a UV-Vis spectrophotometer (205 nm) at different time intervals. The *in vitro* drug release test revealed that the release rate of 5-ASA in buffer solutions increased with the silica content in the composites; on the contrary, the increase of the content of 3-(trimethoxysilyl)propyl methacrylate (TSPA), a coupling agent, decreased the drug release rate.

## 1. Introduction

Mineral clays of the smectite group present a set of structural characteristics that make them attractive for the development of catalysts, sorbents, supports for drug or enzymes, and intercalation of organic molecules [1–5]. Organosilanes are widely used for the modification of silica surfaces. The silylation mechanism for the attachment of organosilane to amorphous silica and alumina surfaces has been commonly reported [6–8]. Recently, organosilanes have been employed for the modification of layered silicates, with smectites and vermiculites being the most commonly clays used to investigate the functionalization processes [9–12]. Organo-functional silanes, represented as RSiX_3_, are often used as coupling agents to enhance the adhesion between polymer and silica filler. The methoxy or ethoxy groups, represented by X, can be hydrolyzed with water. The silanol groups of hydrolyzed coupling agents can be condensed with other silanol groups on a glass or ceramic surface. The organo-functional group, R, can be bonded chemically to a polymer matrix. Thus, the adhesion between polymer and inorganic fillers was improved due to the chemical bonds that formed at the interface. To our best knowledge, how the interface inside organic–inorganic composite materials as well as the coupling agents affect the drug release property has not been studied. Understanding the interfacial interactions and structure is important to better design and application of organic–inorganic composite drug delivery systems.

Perlite is an attractive material as drug carriers because of its stability over a fairly wide range of pH (excluding alkaline), relative inertness in many environments, and transparency in the UV-visible spectrum. Perlites are amorphous aluminum silicates with high content of silica more than 70%. Commercially, the term perlite is used describe either natural or expanded perlite which formed by heating quickly [13]. Inorganic support materials including silica gels, alumina, zeolite and perlite are focused due to their thermal and mechanical stability, no-toxicity and high resistance against environment pH [14]. Lots of inorganic support materials, however, have too expensive cost because of being synthesized from organism-silicon compounds, such as recently developed materials MCM-41, SBA-15, meso-cellular foams [15]. Here the advantage of perlite instead of the other supports is more inexpensive than the other supports [16].

## 2. Results and Discussion

**Scheme 1.**
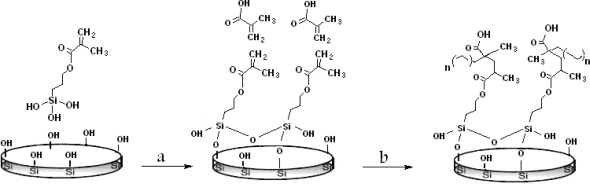
Surface modification of perlite: (a) silylation with TSPA and (b) graft polymerization with MAA.

In the present study perlite was selected for drug delivery support. Organic/inorganic composite were synthesized by graft copolymerization of MAA onto TSPA-modified silica particles (variable feed ratio as shown in Table 1) in a solution of ethyl acetate (Scheme 1). In this step, the formation of grafted polymer chains is typically attributed to both propagation of growing surface chains (surface propagation) and coupling termination between growing homopolymer chains and growing surface chains (polymer grafting) [17].

5-aminosalicylic acid (5-ASA) is useful for localized chemotherapy of inflammatory bowel disease (IBD), but this drug is likely to be absorbed or degraded in the stomach and small intestine before reaching the colon sites [18]. 5-ASA was loaded into these organic/inorganic composite and *in vitro* release profiles were established separately in enzyme-free simulated gastric and intestinal fluids (SGF and SIF, respectively). Influences of different factors, such as content of MAA and swelling were studied.

Grafting the polymer above 70 °C was not feasible due to a rapid volatilization of the ethyl acetate solvent (bp = 77 °C) accompanied by a relatively high rate of conversion. Each grafting experiment was performed by adding TSPA-modified particles into a predetermined volume of MAA/ethyl acetate solution. The slurry mixture was then heated to a desired temperature followed by the addition of an appropriate amount of the AIBN initiator.

### 2.1. Analyses of Extent Interactions between Organic and the Inorganic Moieties

It is important to know whether, and to what extent, the reaction between the silica particles and the polymer matrix has occurred. Therefore, THF extractions of PMAA/perlite composite were performed for 10 days to evaluate the interaction between organic and inorganic phases. If there were no strong interactions between PMAA and the inorganic moieties, THF, a good solvent for PMAA would dissolve the polymer and leave the inorganic component whose weight will almost equal to the amount of silica used in the composites. The resulting values of the residue percentage were listed in Table 2. When 5% (v/v) TSPA was introduced, the residue jumped to 88% and increased with increasing amount of TSPA (10% (v/v)) in composites (97%). That is to say, with the existence of TSPA in the composites, strong chemical bonds were formed between polymer and silica particles. The crosslinking between organic and inorganic phases was strengthened, and silica particles were not easily “drop off” from composites with TSPA.

### 2.2. Thermal Behavior

The thermal behavior of polymer composites is important in relation to its properties for controlling the release rate in order to have a suitable drug dosage form. Differential scanning calorimetry (DSC) was used for analysis of composites. The glass transition temperature (Tg) was determined from the DSC thermograms. As shown in Table 2, the higher Tg values are probably related to the presence of silica particles as well as the coupling agent TSPA, which would decrease the flexibility of the composites and the ability of the composites to undergo segmental motion, which would increase the Tg values.

Diffusion rate of small molecules through polymer matrix is often lowered with the increasing Tg for the increasing restriction of chain-segment mobility. Since 5-ASA is also a small molecule, Tg may influence the 5-ASA release behaviors of PMAA/perlite composites. However, the Tg of each composite in this study is above 150 °C, which is much higher than the temperature of release study. Therefore, the effect of Tg difference may be less important for drug release study.

### 2.3. Swelling of Composite

The swelling value of organic/inorganic composite in pH 1 and pH 7.4 at 37 °C for 2 days are given in Table 3. In this study, the swelling ratio (SR) of the prepared composite samples was determined in order to know whether SR would influence their drug release properties [19,20]. It has been reported that in organic/inorganic composite, water-uptake mainly occurs in the organic phase matrix [21]. In these composite, an increase in the content of MAA in the feed monomer mixtures resulted in less swelling in SGF but greater swelling in SIF. The loading numbers in Table 3 shows which that existence of polar functional groups such as carboxylic acid are needed not only for loading drug on the polymer but also for pH-sensitive properties of the polymer. The hydrogen-bonding and electrostatic interactions increased with MAA content in the copolymer networks. Because the increase of MAA content in the hydrogels provides more hydrogen bonds at low pH and more electrostatic repulsion at high pH.

Use of TSPA remarkably reduced equilibrium water uptake for TSPA-modified samples. This reduction should be attributed to the improved interfacial adhesion that avoided an easy penetration of water molecules into the modified composites and reduced water accumulation in interfacial voids [22,23]. With increased cross-linking and an increase in the reticulated degree of the polymer, diffusion of the water in the network’s polymer is reduced and the swelling is slower.

### 2.4. Release Studies

Although oral delivery has become a widely accepted route of administration of therapeutic drugs, the gastrointestinal tract presents several formidable barriers to drug delivery. Colonic drugs delivery has gained increased importance not just for the delivery of the drugs for the treatment of local diseases associated with the colon but also for its potential for the delivery of proteins and therapeutic peptides. To achieve successful colonic delivery, a drug needs to be protected from absorption of the environment of the upper gastrointestinal tract (GIT) and then be abruptly released into the proximal colon, which is considered the optimum site for colon-targeted delivery of drugs.

Among the various methods that have been developed to assist to these problems [24–30], use of environmentally sensitive hydrogels, especially pH-sensitive carriers, is the most promising method. The objective of this study is to utilize the pH sensitivity composite for oral drug delivery. Then, the grafting of acrylic monomers onto perlite could result in combined properties such as biocompatability, nontoxicity, and higher bioadhesion, which would confer attractive characteristics on the newly prepared composite materials [31].

**Figure 1.**
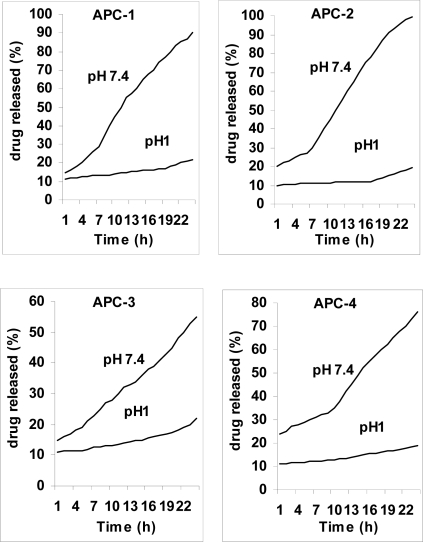
Release of 5-ASA from composites as a function of time at 37 °C.

In order to study potential application of APC containing 5-ASA as a pharmaceutically active compound, we have studied the drug releases behavior of the composite under physiological conditions. Although the composites were not soluble in water, they were dispersed in a buffer solution, and the drug release was evaluated as a heterogeneous system. The percent of released drug from organic/inorganic carriers by different amount of TSPA and MAA as a function of time is shown in Figure 1. The concentration of 5-ASA released at selected time intervals was determined by UV spectrophotometry at 205 nm.

It can be seen that the drug release rate decreased with the increasing content of TSPA. The samples with TSPA had higher crosslinking densities, they had blurred interface between polymer matrix and silica particles, indicating the better adhesion between two phases. And this would decrease diffusion path of drug and leads to slower drug release rate. The other mechanism is the crosslinking effect of the TSPA to the polymer matrix. Besides the improvement of interfacial adhesion between polymer matrix and silica, TSPA has also crosslinking effect to the polymer chains. With higher TSPA content, the mobility of the polymer chains were limited, this may result in decreased diffusion rate of small molecules like drug within the polymer matrix. This might be the reason why the drug release of samples with 5% and 10% coupling agent were different while their SR values were nearly equivalent according to Figure 1.

As the content of MAA in the feed monomers increased, hydrolysis rate decreased at pH 1 but increased at pH 7.4. This was because a higher MAA content in the polymer networks led to higher carboxylate anion concentration at high pH. In other words, the existence of hydrogen-bonding interactions between –COOH groups in the polymer matrix results in a complex structure within the network, and so the movement of polymeric segments is restricted. In other hand, Figure 1 clearly shows a release amount at the beginning of the release process at pH 1 is approximately 10%. In drug loading, adsorption of drug molecule can be occurred in and on the composites. It has been established that the release of adsorb drug molecules on composite surface is very fast.

This also accounts for minimum hydrolyzing of the gel in a medium of pH 1. However, when the sample is placed in a medium of pH 7.4, the almost complete ionization of –COOH groups present within the polymer network not only increases the ion osmotic swelling pressure to a great extent but also enhances the relaxation of macromolecular chains because of repulsion among similarly charged –COO^−^ groups. These two factors ultimately result in a greater increase in the water uptake. In pH 7.4 with completed ionization and an increase in the hydrophilicity of the polymer, diffusion of the hydrolyzing agents on polymer is increased and the hydrolysis rate increased [32].

## 3. Experimental

Surface graft polymerization of MAA onto perlite particles was carried out via a two-step process: surface activation (TSPA) followed by graft polymerization of MAA in ethyl acetate with 2,2’-azobisisobutyronitrile (AIBN) initiator.

### 3.1. Materials

3-(Trimethoxysilyl)propyl methacrylate (TSPA, 99%) was purchased from Sigma-Aldrich (St. Louis, MO, USA). Methacrylic acid (MAA) and 2,2′-azobisisobutyronitrile (AIBN) were purchased from Merck (Darmstadt, Germany). All other chemicals were of analytical grade and were purchased from Fluka (Tokyo, Japan). Perlite obtained from Kan Azar Tabriz Co. (Tabriz, Iran). Methacrylic acid was purified by distillation under vacuum. Initiator of 2,2′-azobisisobutyronitrile (AIBN) was purified by crystallization from methanol.

The IR spectra were recorded on a Shimadzu FT IR-408 spectrophotometer. The DSC curves were obtained on a TGA/SDTA 851 calorimeter at heating and cooling rates of 10 °C/min under N_2_. The amount of released drug was determined on a Philips PU 8620 UV spectrophotometer at the absorption maximum of the free drug in aqueous alkali (λmax = 205 nm) using a 1 cm quartz cell. The HPLC apparatus (Bischoff, Germany) consisted of Bruker LC-21, equipped with a Bruker UV-Vis detector model LC 313 I, Rheodyne loop injector and a C18 reversephase column of Spherisorb-CN (250 × 4.6 mm id., particle size 5 μm).

### 3.2. Silylation

After separating fine perlite powder of special mesh size with a sieve, 25 g of dry perlite were washed by stirring overnight in methanol to remove any organic contaminant and then washed by distilled water. After that 5N NaOH was added to the cleaned perlite and the solution was heated for 30 min in a boiling water bath. Precipitated perlite were filtered and rinsed with excess water until NaOH removed and the washed water reached pH 7. Perlite powder was suspended in two freshly prepared solution of TSPA (5 and 10% (v/v) in 50 mM acetate buffer, pH 4.0. The suspensions were incubated at 75 °C for 4 h with constant mixing then washed thoroughly with abundant water in order to remove TSPA molecules not linked to the surface of support. IR (KBr): 1,730, 1,640, 1,455, 1,260, 1,087, 817 cm^−1^.

### 3.3. Graft Polymerization

Organic/inorganic composite were synthesized by graft copolymerization of MAA onto TSPA-modified silica particles (variable feed ratio as shown in Table 1) in a solution of ethyl acetate. Copolymerization was carried out in the presence of 2,2′-azobisisobutyronitrile (AIBN) as an initiator (0.02 mol·L^−1^) at 60–70 °C in a thermostatic water bath. All experiments were carried out in Pyrex glass ampoules sealed off under vacuum. The conversion of monomer into polymer was determined by UV spectral analysis. After the desired time (24 h) the composites was collected, washed with deionized water for one day and the water was changed every six hours in order to remove any unreacted monomers. After washing, the samples were dried in air and stored in desiccators until use. IR (KBr): 3,350–2,550 (broadened, −COOH group), 1,725, 1,456, 1,258, 1,119, 890 cm^−1^.

### 3.4. The Swelling Ratio of Matrices

In a typical test, the films after having been soaked in various buffer solutions (pH 7.4 and pH 1) at 37 °C for two days were weighed after excess water was wiped off from the film surface with filter paper. The swelling ratio (SR) of matrices was calculated using the following equation:

(1) SW (%)=[(Ws − Wd)/Wd]×100

where Ws and Wd represent the weight of swollen and dry samples, respectively.

### 3.5. Drug Loading in Composites

Subsequently, 100 mg of each matrix was placed in 10 mL of 5-aminosalicylic acid (5-ASA) (10 mg·mL^−1^) to suck up the total amount of the drug solution. After approximately 6 h, the swollen composits loaded with drug were placed in desiccators and dried under vacuum at room temperature.

### 3.6. Determination of the Amount of Drug Entrapped

The amount of drug entrapped in the composite was determined by an indirect method. After the gel preparation, the washings were collected, filtered and tested using UV–VIS spectroscopy. The difference between the amount of drug initially employed and the drug content in the washings is taken as an indication of the amount of drug entrapped. The values of quantification of entrapped drug in the composite based on the total amount are given in Table 3.

### 3.7. Characterization of Hydrolysis Product

Composite-drug adduct (90 mg) was dispersed in 20 mL of pH 8 buffered solution. The reaction mixture was maintained at 37 °C. After 24 h the hydrolysis solution was sampled and neutralized with 1 M HCl and the solvent was evaporated in vacuum. The resulting crude product was treated with 20 mL of mixture (1:1) of acetone: H_2_O and heated. The suspension was then filtered and the solution was evaporated under reduced pressure. Samples were measured using HPLC-UV. The column used was ODS (C18) and isocratic elution was performed using 50% methanol and 50% buffer containing 0.05 M NH_3_. The flow-rate and injection volume were 1 mL/min and 100 μL, respectively. 5-ASA was detected at a retention time of 2.4 min.

### 3.8. In Vitro Drug Release Study

Each drug-loaded composite sample was soaked in 5 mL of aqueous buffer solution (SGF: pH 1 or SIF: pH 7.4). The mixture was introduced into a cellophane membrane dialysis bag. The bag was closed and transferred to a flask containing 20 mL of the same solution maintained at 37 °C for 24 h. The external solution was continuously stirred, and 3 mL samples were removed at selected intervals. 5-ASA content in the buffer solutions was measured by UV-Visible spectrophotometer at the wavelength λ_max_ = 205 nm. All the drug release studies were carried out in triplicate.

## 4. Conclusions

In this study, PMAA/perlite composite materials with various TSPA proportion and perlite content have been prepared via sol–gel process. The influence of TSPA proportion and MAA content on their drug release properties has been evaluated using 5-ASA as a model drug. The swelling test supported the hypothesis that the interface between the polymer matrix and silica particles played a key role in drug release property of the composite system. The drug release rate increased with the increasing content of perlite while decreased with the amount of coupling agent in the composite samples. The drug release rate of this composite system can be modulated by adjusting the content of both inorganic fillers and the coupling agent.

## Figures and Tables

**Table 1.** The molar composition of composite.

**Organic/inorganic composite**	**Molar composition in the feed (%)**	
	**TSPA**	**MAA**
APC-1	5	10
APC-2	5	30
APC-3	10	10
APC-4	10	30

**Table 2.** DSC data and THF extraction results.

**Organic/inorganic composite**	**Tg (**°C**)**	**Residue (%)**
APC-1	150	97
APC-2	162	93
APC-3	180	88
APC-4	195	83

**Table 3.** Percent of swelling and drug loading numbers.

**Composites**	**Maximum constant swelling (%) pH 1**	**Maximum constant swelling (%) pH 7.4**	**Percent of 5-ASA-loading (%)**
APC-1	8	26	87
APC-2	5	35	96
APC-3	4	22	78
APC-4	2	30	90
